# Interplay between the Xer recombination system and the dissemination of antibioresistance in *Acinetobacter baumannii*

**DOI:** 10.1093/nar/gkae1255

**Published:** 2025-01-07

**Authors:** Corentin Blanchais, Carine Pages, Manuel Campos, Kenza Boubekeur, Rachel Contarin, Mathias Orlando, Patricia Siguier, Maria-Halima Laaberki, François Cornet, Xavier Charpentier, Philippe Rousseau

**Affiliations:** Laboratoire de Microbiologie et Génétique Moléculaires, Centre de Biologie Intégrative, Université de Toulouse, CNRS, UPS, 165 Rue Marianne Grunberg-Manago, 31400 Toulouse, France; Centre International de Recherche en Infectiologie, INSERM, U1111, Université Claude Bernard Lyon 1, CNRS, UMR5308, École Normale Supérieure de Lyon, Université de Lyon, 46 All. d'Italie, 69007 Lyon, France; Laboratoire de Microbiologie et Génétique Moléculaires, Centre de Biologie Intégrative, Université de Toulouse, CNRS, UPS, 165 Rue Marianne Grunberg-Manago, 31400 Toulouse, France; Laboratoire de Microbiologie et Génétique Moléculaires, Centre de Biologie Intégrative, Université de Toulouse, CNRS, UPS, 165 Rue Marianne Grunberg-Manago, 31400 Toulouse, France; Laboratoire de Microbiologie et Génétique Moléculaires, Centre de Biologie Intégrative, Université de Toulouse, CNRS, UPS, 165 Rue Marianne Grunberg-Manago, 31400 Toulouse, France; Laboratoire de Microbiologie et Génétique Moléculaires, Centre de Biologie Intégrative, Université de Toulouse, CNRS, UPS, 165 Rue Marianne Grunberg-Manago, 31400 Toulouse, France; Laboratoire de Microbiologie et Génétique Moléculaires, Centre de Biologie Intégrative, Université de Toulouse, CNRS, UPS, 165 Rue Marianne Grunberg-Manago, 31400 Toulouse, France; Laboratoire de Microbiologie et Génétique Moléculaires, Centre de Biologie Intégrative, Université de Toulouse, CNRS, UPS, 165 Rue Marianne Grunberg-Manago, 31400 Toulouse, France; Centre International de Recherche en Infectiologie, INSERM, U1111, Université Claude Bernard Lyon 1, CNRS, UMR5308, École Normale Supérieure de Lyon, Université de Lyon, 46 All. d'Italie, 69007 Lyon, France; VetAgro Sup, Université de Lyon, 1 avenue Bourgelat, 69280 Marcy-l'Etoile, France; Laboratoire de Microbiologie et Génétique Moléculaires, Centre de Biologie Intégrative, Université de Toulouse, CNRS, UPS, 165 Rue Marianne Grunberg-Manago, 31400 Toulouse, France; Centre International de Recherche en Infectiologie, INSERM, U1111, Université Claude Bernard Lyon 1, CNRS, UMR5308, École Normale Supérieure de Lyon, Université de Lyon, 46 All. d'Italie, 69007 Lyon, France; Laboratoire de Microbiologie et Génétique Moléculaires, Centre de Biologie Intégrative, Université de Toulouse, CNRS, UPS, 165 Rue Marianne Grunberg-Manago, 31400 Toulouse, France

## Abstract

Antibiotic-resistant infections are a pressing clinical challenge. Plasmids are known to accelerate the emergence of resistance by facilitating horizontal gene transfer of antibiotic resistance genes between bacteria. We explore this question in *Acinetobacter baumannii*, a globally emerging nosocomial pathogen responsible for a wide range of infections with a worrying accumulation of resistance, particularly involving plasmids. In this species, plasmids of the Rep_3 family harbor antibiotic resistance genes within variable regions flanked by potential site-specific recombination sites recognized by the XerCD recombinase. We first show that the Xer system of *A. baumannii* functions as described in *Escherichia coli*, resolving chromosome dimers at the *dif* site and recombining plasmid-carried sites. However, the multiple Xer recombination sites found in Rep_3 plasmids do not allow excision of plasmid fragments. Rather, they recombine to cointegrate plasmids, which could then evolve to exchange genes. Cointegrates represent a significant fraction of the plasmid population and their formation is controlled by the sequence of recombination sites, which determines the compatibility between recombination sites. We conclude that plasmids in *A. baumannii* frequently recombine by Xer recombination, allowing a high level of yet controlled plasticity in the acquisition and combination of antibiotic resistance genes.

## Graphical Abstract

**Graphical Abstract:**
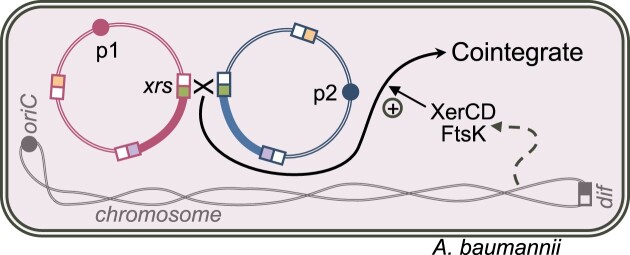

## Introduction

In bacteria, most replicons are circular, and an odd number of homologous recombination events lead to their dimerization, during their replication. This interferes with the even distribution of replicons between daughter cells during cell division. To overcome this problem, prokaryotes have evolved replicon dimer disassembly sites, which we call *xrs* (Xer recombination sites) and which are acted upon by dedicated Xer recombinases (1,2). The importance of this function for faithful replicon segregation explains its high conservation and the Xer system is now considered as one of the most conserved structural features of circular chromosomes in bacteria and archaea (3,4).

**Figure 1.**
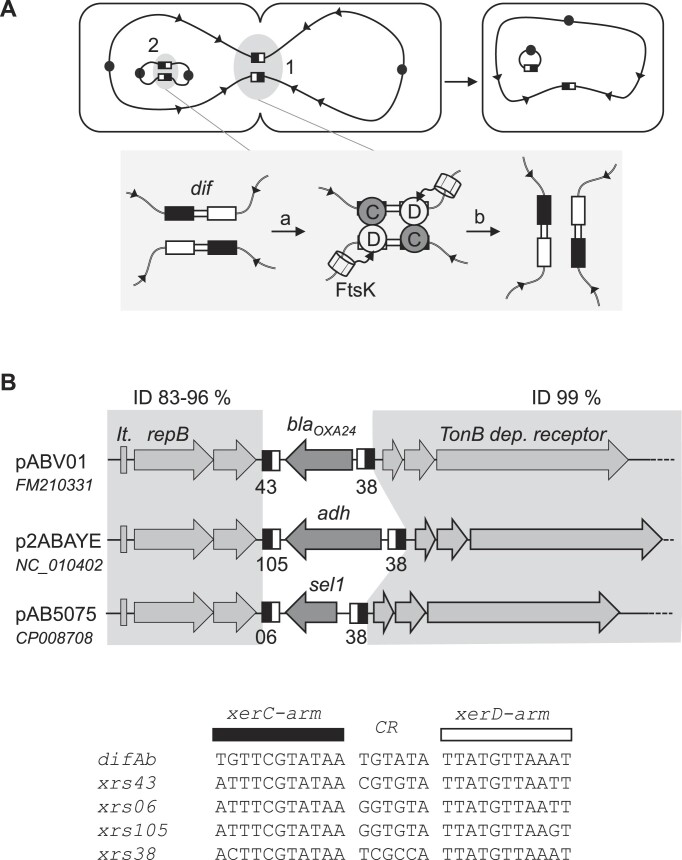
Xer system. (**A**) Xer is a site-specific recombination system that resolves chromosome dimers prior to cell division (1). Xer is hijacked by plasmids to resolve their dimers and ensure their correct vertical transmission (2). Xer recombination sites (*xrs*) are shown as black and white boxes. The *xrs* present on the chromosome is called *dif*. Black circles represent origins of replication. The *xrs* consist of a XerC binding arm (black rectangle) and a XerD binding arm (white rectangle) separated by a central region (CR). XerC (gray circle) and XerD (white circle) bind *dif* (or *xrs*) and form a recombination complex (a) within which, once activated by FtsK, they will catalyze the successive exchange of the two DNA strands of the central region (b) to form the recombinant DNA. (**B**) Some *Ab* plasmids carry multiple *xrs* and are highly identical (ID), except for regions flanked by *xrs* (17). The first conserved block contains replication functions: repB and iterons (It.). The second conserved block contains a TonB-dependent receptor gene. The xrs flanking the variable genes are shown with their sequences aligned.

The XerCD recombination process is particularly well described for the unique *xrs* located on bacterial chromosomes, known as the *dif* site (Figure 1A). The *dif* site consists of two protein-binding arms separated by a central region. XerC and XerD bind specifically to their respective binding arms (5) to form a recombination complex. Within this complex, a pair of recombinases, either XerC or XerD, catalyzes a nucleophilic attack on the DNA and forms a covalent bond with the *dif* site. Strands are then exchanged between the two *dif* copies in the central region to form a Holliday junction. The second pair of recombinases then cuts and exchanges the second pair of strands, completing the recombination reaction (5–7).

To recombine the chromosomal *dif* site, XerD first cleaves, when activated by FtsK, a division septum-associated DNA translocase, which is essential for cell division (8–10). FtsK translocates to *dif* by recognizing KOPS sequences oriented to *dif* on the chromosome (11–13). Upon arrival at the *dif* site, FtsK activates recombination through a specific contact with XerD (9). This FtsK control of the XerCD-*dif* recombination allows spatiotemporal control of chromosome dimer resolution: at the midcell during septation.

Plasmids are also affected by this dimerization problem (14). A number of them use the Xer system to resolve their dimeric forms and some have evolved *xrs* to be independent of the bacterial cell cycle [e.g.*cer* or *psi* are FtsK-independent *xrs* (15)].

While it is expected that plasmids carry a single *xrs* site to resolve dimers (15), an increasing number of *Acinetobacter baumannii* plasmids carry multiple *xrs* [called *Re27*, *pdif* or *pXerC/D* (16–20)]. *Acinetobacter baumannii* (Ab) is a human pathogen involved in nosocomial diseases, and the accumulation of *xrs* on its plasmids may be related to its ability to acquire resistance to multiple antibiotics. For example, the pABV01 plasmid contains two *xrs* flanking the *bla_oxa-24_* gene, which confers resistance to carbapenems (17). This plasmid is related to two others (p2ABAYE and pAB0057) that are very similar, except for the region flanked by their two *xrs* (17) (Figure 1B). These observations led to the concept of *xrs* cassettes [adaptive genes flanked by two *xrs*, also called *pdif* modules (18,20,21)], suggesting the involvement of the Xer system in the spread of antibiotic resistance genes. Recent work suggests that *xrs* present on *Ab* plasmids are hotspots of recombination between plasmids (19,22,23) and that they could be processed by Xer recombinases (24). However, very little is known about the activity of these sequences and/or cassettes and whether they are *bona fide* Xer recombination sequences. We present here a functional study of the Xer system of *Ab* on chromosomal (*dif*) and plasmidic *xrs*. Our results demonstrate that Xer catalyzes recombination between *Ab* plasmids via an original cointegration/resolution mechanism that may explain the rapid spread of antibiotic resistance in this human pathogen.

## Materials and methods

### *Escherichia coli* strains and plasmids

The *xrs* cassette carrying plasmids were derived from pFX346 (25). Tandems of *xrs* (direct or inverted orientation depending on the case) separated by a SalI site were first obtained by hybridization of two complementary oligonucleotides and inserted into pFX346 (between BamHI and SphI). *lacIq* was then inserted between *xrs* (SalI) to give pROUT29 (*dif_Ab_*-*lacIq*-*dif_Ab_*), pROUT25 (*xrs43*-*lacIq*-*xrs38_inv_*), pROUT31 (*xrs105*-*lacIq*-*xrs38_inv_*) and pROUT32 (*xrs6*-*lacIq*-*xrs38_inv_*). *Escherichia coli* strains were derived from the K12 strain LN2666. The CP1518 strain was derived from CP109 (LN2666 Δ*dif*::Tc, Δ(*lacI*), *xer*::Gm) (26), by insertion–excision of a *dif_Ab_-lacI-dif_Ab_* cassette into the Tc gene (27). For this modification, the *dif_Ab_-lacI-dif_Ab_* cassette was first inserted between BamHI and SpaI sites within plasmid pLN135 to obtain pCP179. The *recF*::Kn allele [KEIO collection (28)] was introduced (P1 transduction) into CP1088 [LN2666 Δ*xerD*, Δ*xerC* (29)] to obtain CB50 (LN2666 Δ*xerD*, Δ*xerC*, *recF*::Kn). *Acinetobacter baumannii xer* genes (*xerC_Ec_, xerC_Ab_, xerD_Ec_, xerD_Ab_, xerC_RQ_, xerD_RQ_* and *xerDγ_ftsK_*) were ordered from GenScript company. They were cloned into pET32 to obtain production plasmids pROUT26 (*xerC_Ab_*), pROUT27 (*xerD_Ab_*), pCB01 (*xerC_Ab_*-R254Q), pCB02 (*xerD_Ab_-R255Q*) and pCB03 (*xerDγ_ftsK_*). These genes were also used to construct pSC101-based *in vivo* expression vectors. The pBAD promoter (pBAD18) was cloned into pSC101 plasmid and the *xer* genes were cloned alone, or in operon, under the control of their native promoter to obtain pLN1xerCD_Ec_, pLN1*xerCD_Ab_*, pLN1*xerCDγ_Ab_* and pLN1*xerCD_RQ_*.

### *Acinetobacter baumannii* strains and plasmids

Ab5075-T and AYE-T are *A. baumannii* stains derived from AB5075-UW (NZ_008 706) and AYE (NC_010410) (30). To obtain CB-AB01 (Ab5075-T *xerC*::*sacB_aacC*) and CB-AB03 (ABAYE-T *xerC*::*sacB_aacC4*), AYE-T and AB5075-T were transformed with DNA fragments carrying a *sacB_aacC4* gene flanked by two 2-kb homology regions. This DNA fragment was obtained by overlap extension polymerase chain reaction (PCR) of three DNA fragments using oligonucleotides 74–75, 78–79 and 76–77 (Supplementary Table S1). Transformants were plated on LB agar supplemented with 30 μg/ml apramycin. These strains were then transformed with a DNA fragment containing the two 2-kb homologies (oligonucleotides 74–80 and 78–81; Supplementary Table S1) to obtain ABAYEΔ*xerC* and AB5075Δ*xerC* deletion mutants. Transformants were selected on M9 agar supplemented with 10% sucrose (30). The plasmids used in this work are pABV01 (FM210331), p1ABAYE (NC_010401), p2ABAYE (NC_010402) and p2AB5075 (CP008708).

### *In vitro* experiments

Xer proteins were purified as described previously (25,29). For electrophoretic mobility shift assay (EMSA) experiments, 28-bp 5′-end-labeled [CY3] DNA fragments carrying *dif_Ab_* or *xrs* sites were obtained by hybridization of complementary strands (Supplementary Table S1). EMSA reactions were performed as previously described (25,29,31) and analyzed using a typhoon TRIO GE. Cleavage assays were performed as previously described (32) (Supplementary Table S1). DNA substrates were prepared by hybridization of different oligonucleotides (Supplementary Table S1) and the assay results were analyzed by sodium dodecyl sulfate–polyacrylamide gel electrophoresis (SDS-PAGE; Mini-PROTEAN TGX gels, 4–20%).

### *In vivo* recombination assay

For the chromosome-based deletion assay, *E. coli* CP1518 was transformed with pLN1-type expression vectors (pLN1*xerCD_Ec_*, pLN1*xerCD_Ab_*, pLN1*xerCDγ_Ab_* and pLN1*xerCD_RQ_*). Serial dilutions of the transformants were plated on LB agar medium supplemented with 5 μg/ml chloramphenicol, 40 μg/ml X-Gal and 0.4% arabinose or glucose and grown overnight at 37°C as previously described (31).

For the plasmid-based deletion assay, CB50 (LN2666 Δ*xerD*, Δ*xerC*, *recF*::Kn) was transformed with pROUT29, pROUT25, pROUT31 or pROUT32, together with a pLN1-based *xer* expression plasmid. Transformants were plated on LB agar supplemented with 5 μg/ml chloramphenicol and 25 μg/ml ampicillin. After overnight cultivation in selective LB medium containing 0.4% arabinose, plasmid content was extracted (QIAprep Spin Miniprep Kit) and analyzed by 1% agarose gel electrophoresis.

### Cointegrate detection and quantification

Cointegrates were detected by PCR (EmeraldAmp GT PCR Master Mix) on plasmid DNA matrices purified from *A. baumannii* strains (QIAprep Spin Miniprep Kit). Pairs of oligonucleotides used to detect non-recombined plasmids were 94–105 for *xrs105*, 95–101 for *xrs38* and 103–121 for *xrs10*. The expected lengths of the amplicons were 1.3, 1.0 and 1.2 kb, respectively. The oligonucleotide pairs used to detect cointegrates were 94–117 for *xrs6/105* recombination, 95–119 for *xrs66/38* recombination and 103 + 120 for *xrs107/10* recombination. The expected amplicon lengths were 0.8, 0.7 and 1.2 kb, respectively. PCR products were analyzed by 1% agarose gel electrophoresis.

Quantification of cointegrates was performed using digital droplet PCR (ddPCR; Bio-Rad QX200 droplet reader). Oligonucleotides were designed by using the Primer3Plus program (Supplementary Figure S1). DNA matrix was extracted from 1 ml of OD_600_ = 0.6 cultures (Wizard Genomic DNA Purification Kit). DNA was then digested with EcoRI (FastDigest, Thermo Fisher) and diluted 100-fold. A typical 20 μl ddPCR reaction contains 1 μl of DNA matrix, 2× EvaGreen ddPCR Supermix (Bio-Rad) and 50 nM of each primer. A typical amplification reaction is as follows: 5 min at 95°C; 40×: 30 s at 95°C; 30 s at 58°C; 1 min at 72°C. A 2.5°C/s ramp rate was used. Results were analyzed using QuantaSoft droplet reader software.

## Results

### Characterization of the Xer system of *A. baumannii*

**Figure 2.**
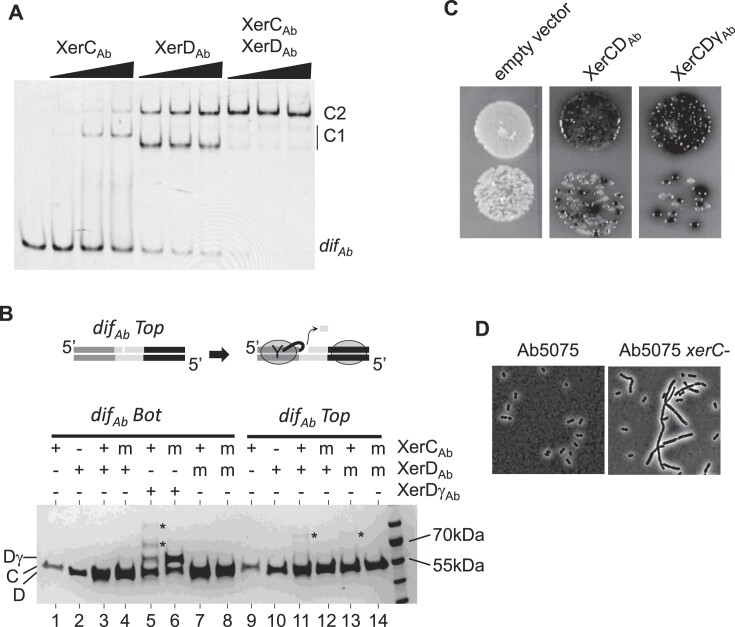
Activity of the Xer system of *Ab*. (**A**) EMSA of *dif_Ab_* with XerCD_Ab_. The DNA fragment (*dif_Ab_*) is 56 bp long and is 5′-CY3. XerC_Ab_ (μM) = 0, 0.48, 1.2 and 2.4. XerD_Ab_ (μM) = 0, 0.64, 1.6 and 3.2. (**B**) SDS–PAGE analysis of suicide substrate assays (top diagram). DNA molecules (*dif_Ab_* top or bottom nicked) are 10 μM, whereas proteins are 2 μM. XerC_Ab_ is indicated as C, XerD_Ab_ as D, XerDγ_Ab_ as Dγ and covalent products formed between DNA and proteins as *. Mutant proteins (m) were used as negative controls. XerC-R254Q and XerD-R255Q correspond to a mutation in the catalytic domain that allows DNA binding but not catalysis (46). Note that two covalent complexes are observed between *dif_Ab_* and XerDγ_Ab_ (lane 5). Proteins are visualized by Coomassie Blue staining. (**C**) *Escherichia coli* (CP1518, *Δdif::dif_Ab_-lacI-dif_Ab_*, *Δ(lacI)*, *xer::Gm*) was transformed with vector expressing *xerCDAb*, or *xerCD*γ_Ab_. Serial dilutions of the transformant were plated on solid media supplemented with X-Gal and arabinose (see the ‘Materials and methods’ section). Blue colonies correspond to Xer-induced deletion of the *lacI* gene. (**D**) Photomicrographs of bacteria carrying or not carrying a deletion of *xerC*. Strains were observed by phase contrast microscopy (see the ‘Materials and methods’ section).

First, we investigated the functionality of the *Ab* Xer system (Xer_Ab_). To this end, full-length XerC and XerD proteins from strain AB5075 (hereafter referred to as XerC_Ab_ and XerD_Ab_) were purified in a heterologous system. Binding of the purified proteins to their expected substrate, *dif_Ab_*-containing DNA, was assessed by EMSA (Figure 2). Increasing concentrations of XerC_Ab_ resulted in two shifted bands (C1 and C2). Based on previous observations in the *E. coli* Xer system (5), we deduced that these bands correspond to XerC_Ab_ bound to its binding site (C1) and to both XerC_Ab_ and XerD_Ab_ binding sites (C2). Similar results were obtained for XerD_Ab_. XerD_Ab_ seemed to bind *dif_Ab_* with higher affinity than XerC_Ab_. The C1 complexes migrate differently depending on the protein used, suggesting different conformations of these complexes [e.g. DNA bending (25)]. When both XerC_Ab_ and XerD_Ab_ were added, only a C2-like complex was observed. We inferred that this complex corresponds to the binding of both XerC_Ab_ and XerD_Ab_ to their binding sites on *dif_Ab_*. Taken together, these results show that Xer recombinases of *Ab* form a tripartite complex with *dif_Ab_*, as described for the *E. coli* system (5,29).

We then tested whether XerC_Ab_ and XerD_Ab_ could catalyze the first step of DNA recombination on *dif_Ab_*, i.e. DNA cleavage by the catalytic tyrosine residue, leading to the formation of a covalent bond between the cleaved DNA and the Xer protein. For this purpose, we used a ‘suicide assay’ in which the covalent DNA–protein intermediate is trapped [see the ‘Materials and methods’ section (32) and Figure 2B]. We observed that XerC_Ab_ cleaved *dif_Ab_*-containing DNA when in the presence of XerD_Ab_ (Figure 2B, lanes 9–11). This cleavage was not observed when a catalytic residue of XerC_Ab_ was mutated (lane 12). In contrast, XerD_Ab_ did not cleave *dif_Ab_*, even if in the presence of XerC_Ab_ (Figure 2B, lanes 1–3). This latter result suggests that, as in *E. coli*, activation by FtsK is required for cleavage by XerD_Ab_ (9). As expected, C-terminal fusion of the AB5075 FtsKγ-domain to XerD, designated XerDγ_Ab_, allows cleavage of *dif_Ab_*-containing DNA (Figure 2B, lane 5). Surprisingly, two covalent complexes were observed with XerDγ_Ab_, which could be due to different DNA conformations in the covalent product.

To further characterize the Xer_Ab_ system, we replaced the *dif_Ec_* site on the *E. coli* chromosome with a *dif_Ab_-lacI-dif_Ab_* cassette in a strain deleted for the *xerC* and *lacI* genes [see the ‘Materials and methods’ section (26)]. The resulting strain produced white colonies when plated on LB in the presence of the β-galactosidase substrate (X-Gal), with LacI effectively repressing the β-galactosidase encoding gene *lacZ* (Figure 2C, empty vector). When this strain was transformed with a plasmid expressing both *xerC_Ab_* and *xerD_Ab_* genes, blue colonies appeared, indicating that recombination events had occurred (Figure 2C and Supplementary Table S2). Recombination was 5-fold more efficient when *xerDγ_Ab_* was substituted to *xerD_Ab_* (Figure 2C and Supplementary Table S2), further demonstrating that Xer_Ab_ recombination is controlled by FtsK.

To provide evidence for Xer*_Ab_* functionality in *Ab* replication, we deleted the *xerC_Ab_* gene in strain AB5075. The resulting strain formed filaments (Figure 2D), similar to observations in *E. coli* strains inactivated for chromosome dimer resolution (33).

Taken together, these results demonstrate that the *Ab* XerCD/*dif* system is canonical and behaves as a site-specific recombination system involved in chromosome dimer resolution under the control of the division protein FtsK.

### *Acinetobacter* *baumannii* plasmids carry multiple Xer recombination sites

**Figure 3.**
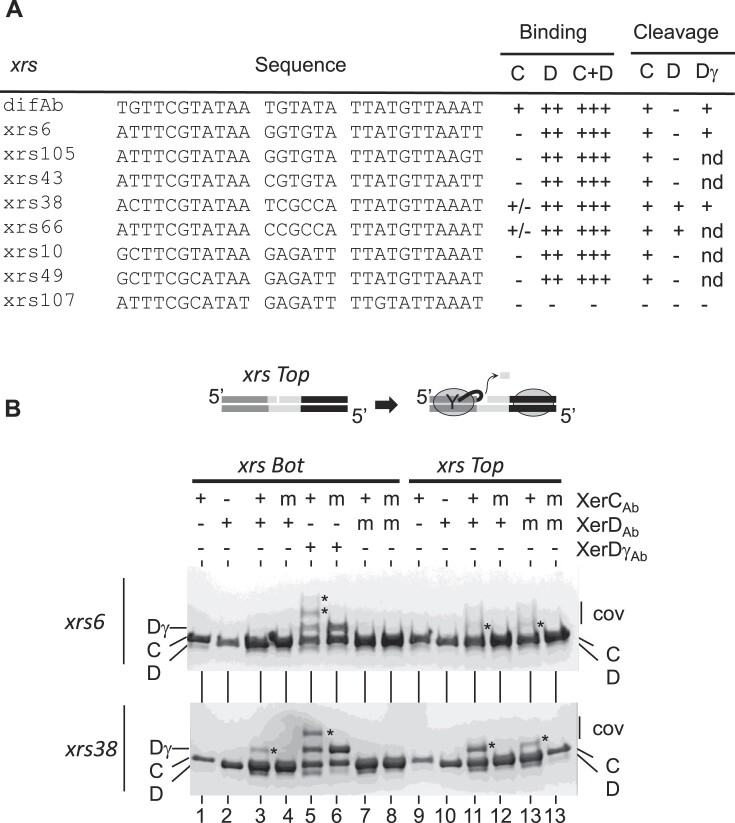
Activity of *xrs* sequences present on *Ab* plasmids. (**A**) Summary of EMSA and cleavage assays performed on different *xrs*. pABV01 contains *xrs38*, *xrs43* and *xrs10*. P1ABAYE contains *xrs*06, *xrs107* and *xrs66*. P2ABAYE contains *xrs38*, *xrs105* and *xrs49*. P2AB5075 contains *xrs38*, *xrs06* and *xrs49*. Experiments were performed as shown in Figure 2. Raw data are shown in Supplementary Figures S1 and S2. (**B**) SDS–PAGE analysis of cleavage assays (top diagram) on *xrs6* and *xrs38*. DNA molecules (*xrs* top or bottom nicked) are 10 μM, while proteins are 2 μM. XerC_Ab_ is indicated as C, XerD_Ab_ as D, XerDγ_Ab_ as Dγ and covalent products formed between DNA and proteins as *. Mutant proteins (m) were used as negative controls. XerC-R254Q and XerD-R255Q correspond to a mutation in the catalytic domain that allows DNA binding but not catalysis (46). Note that two covalent complexes are observed between *xrs6* and XerDγ_Ab_ (lane 5, top gel). Note that the covalent product XerD_Ab_-*xrs38* migrates like XerDγAb (lane 3, bottom gel). Proteins are visualized by Coomassie Blue staining.

We next investigated whether the *xrs* found in multiple copies in *Ab* plasmids are active. We examined a set of *xrs* from three related plasmids of the Rep_3 family reported in clinical strains: pABV01, p2ABAYE and p2AB5075. The results are summarized in Figure 3A (see Supplementary Figure S1 for raw data). All sequences tested, except *xrs107* that is highly degenerated, were bound cooperatively by XerC_Ab_ and XerD_Ab_. XerC_Ab_ alone bound poorly to these sequences, whereas XerD_Ab_ showed a better apparent affinity. This is consistent with the fact that the predicted XerC binding arms of these *xrs* are more divergent from the *dif* consensus than their XerD binding arms (21). Most *xrs* were cleaved by XerC_Ab_ and XerDγ_Ab_ but not by XerD_Ab_ (Figure 3; *xrs6* is shown as a typical example). We conclude that most *xrs* found in *Ab* plasmids appear to be active for Xer recombination.

Interestingly, *xrs38*, which is the only *xrs* present on the three plasmids (Figure 1B), behaves differently. In fact, we found that *xrs38* is bound and cleaved by XerC_Ab_, but also, and unexpectedly, by XerD_Ab_ (Figure 3B). Similarly, x*rs66*, closely related to *xrs38* and present on p1ABAYE (see below), also exhibits this property. This suggests that *xrs38* and *xrs66* can recombine independently of FtsK, suggesting a different role that will be discussed later.

### The *xrs* cassettes that are found on *A. baumannii* plasmids do not form excisable modules

**Figure 4.**
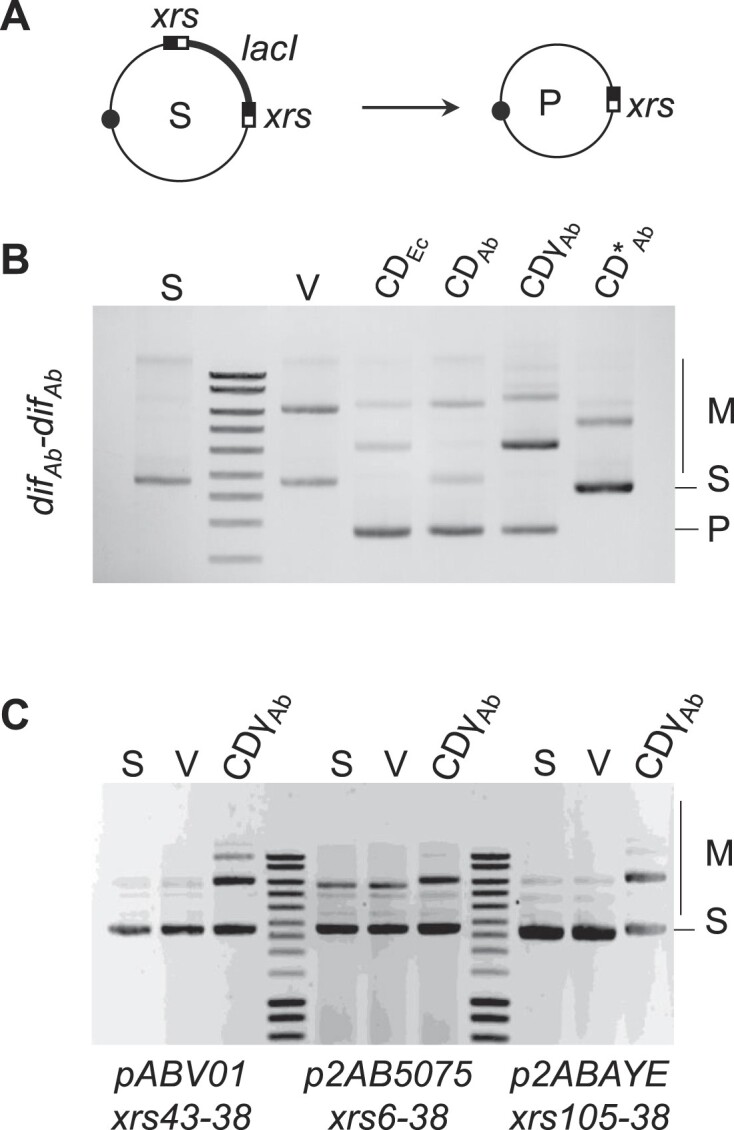
*xrs* cassette deletion. (**A**) The XerCD recombinase, when produced, should delete the *lacI* gene from the substrate plasmid (S), resulting in a deleted plasmid (P) and a non-replicative circle containing *lacI*, which is lost during divisions. Black dots represent the origin of plasmid replication. (**B**) Gel electrophoresis of the plasmid extraction after overnight culture of different *E. coli* strains transformed with a substrate plasmid containing a *dif_Ab_-lacI-dif_Ab_* cassette: S, substrate plasmid used to transform cells; V, plasmids extracted from a strain not expressing any *xer* genes; CD*_Ec_*, plasmids extracted from a strain expressing *xerCD* of *E. coli;* CD*_Ab_*, plasmids extracted from a strain expressing *xerCD* of *Ab;* CDγ*_Ab_*, plasmids extracted from a strain expressing *xerC_Ab_* and a fusion between *xerD_Ab_* and γ domain *of ftsK_Ab_*. Substrate (S) and product (P) plasmids are indicated, as well as multimeric forms of substrate and product plasmids (M). (**C**) Gel electrophoresis of the plasmid extraction after overnight cultivation of different strains transformed with substrate plasmids (S) containing different *xrs-lacI-xrs* cassettes. The *xrs43-38* cassette represents the cassette found in pABV01, the *xrs06-38* cassette represents the cassette found in p2AB5075 and the *xrs105-38* cassette represents the cassette found in p2ABAYE.

Because *xrs* are often found flanking adaptive genes, and because related *Ab* plasmids exhibit variability for sequences flanked by *xrs*, it has been proposed that pairs of *xrs* form a new type of mobile element, or module, that can be exchanged between plasmids (17,18,21). Since in these *xrs* are more palindromic than other *dif*-like sequences described in other bacterial systems (XerC binding arms resemble XerD binding arms) (21), it is not excluded that these *xrs* cassettes could be excised from one plasmid and integrated into another by XerCD_Ab_ recombination. To test this hypothesis, we examined module excision in an *E. coli* strain deleted for *xerC* and *xerD* and expressing different versions of the *xerC* and *xerD* genes from a plasmid (Figure 4A). These strains were transformed with plasmids (S) in which the *lacI* gene is flanked by the tested pair of *xrs*. A deletion product (P) was observed between directly repeated *dif_Ab_* sites in strains expressing *xerCD* genes, either *E. coli* or *Ab* ones (Figure 4B). This shows that *dif_Ab_* can be recombined by XerCD from either *E. coli* or *Ab* and that *E. coli* FtsK can activate XerD_Ab_ (lanes CD_Ab_ and CD_γAB_). No recombination was detected when the strain expressed a mutant (loss of catalytic activity, R255Q) version of *xerD_Ab_* (Figure 4B, lane CD*_Ab_).

We then tested pairs of *xrs* that mimic the genetic organization found in the pABV01, p2AB5075 and p2ABAYE plasmids (Figure 4C). No deletion was detected with any combination of *xrs* cassettes and recombinases. Although most *xrs* cassettes are composed of flanking inverted repeats (16–20), we searched for an *xrs* cassette composed of two directly repeated *xrs*. We found that the *xrs* flanking the *relBE* genes in the pK50b plasmid are directly repeated, and that they were misoriented in the publication (34). We tested the ability of this *xrs* cassette to be deleted from the plasmid by Xer recombination (Supplementary Figure S2). The results show that, as with other *xrs* cassettes, no deletion is observed. This suggests that the *xrs* found in *Ab* plasmids do not form excisable modules and that this is not due to their orientation but rather due to their activity.

Interestingly, this experiment also showed that the expression of *xerCDγ_Ab_* in these strains leads to the formation of multimeric form of the *xrs*-containing plasmids (Figure 4C). This confirms that *xrs* are recognized and recombined *in vivo* by XerCDγ*_Ab_*, leading to recombination between plasmid molecules (intermolecular) rather than within the plasmids (intramolecular).

### High recombination rate between *xrs*-bearing replicons in *A. baumannii*

Xer recombination usually allows excision of a DNA fragment flanked by pairs of *xrs* in direct orientation with an identical central region (2,15). Of note, in *Ab* plasmids, most pairs of *xrs* flanking adaptive genes are often in opposite orientation and/or have divergent central regions (18,19,21). Intramolecular Xer recombination of *Ab* plasmids carrying *xrs* would therefore not be possible. However, as suggested by Balalovski and Grainge in their review of antibiotic resistance gene shuffling in *Acinetobacter*, gene exchange could also occur by recombination between *xrs* carried by coexisting plasmids (20). Intermolecular recombination between *xrs* with closely related central regions would cointegrate the plasmids. Resolution of this cointegration by intramolecular recombination between a second pair of compatible *xrs* would then lead to gene exchange between the recombining plasmids.

**Figure 5.**
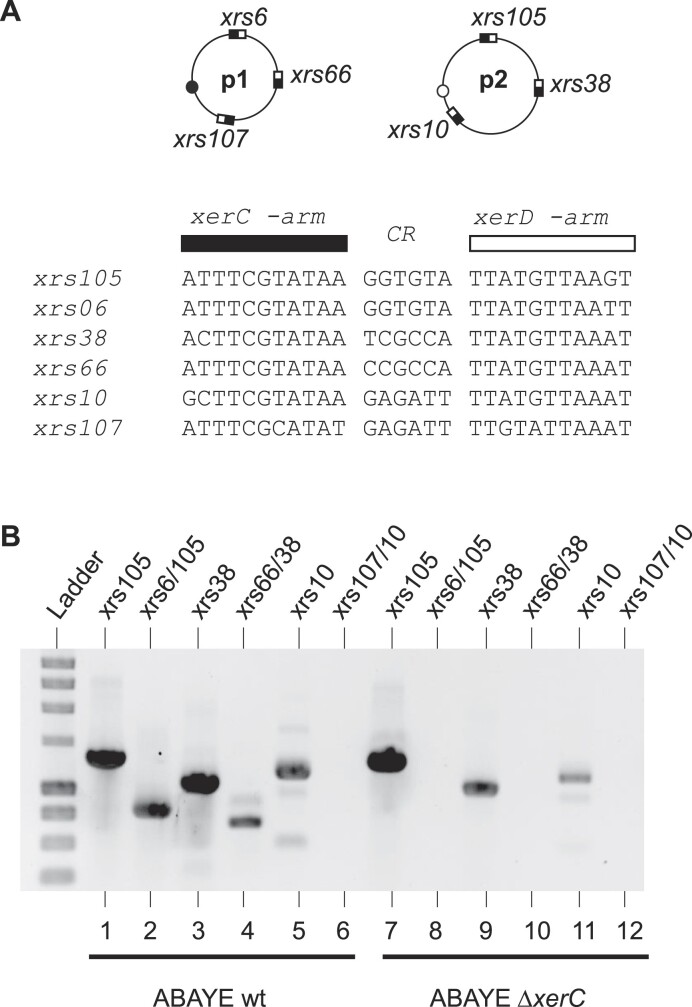
Xer-dependent plasmid recombination. (**A**) Simplified genetic map of p1ABAYE (p1) and p2ABAYE (p2). The *xrs* present on these plasmids are indicated and their sequences are aligned. Black or white dots on plasmid represent the Rep_3 replication origins of these plasmids. (**B**) Gel electrophoresis of the PCR reactions performed on plasmid DNA extracted from wild-type or Δ*xerC* ABAYE strains. The lanes correspond to the amplified *xrs*. For example, lane *xrs105* corresponds to the PCR product obtained when the two primers flank *xrs105* (p2); lane *xrs6/105* corresponds to the PCR product obtained when one primer flanks *xrs6* (p1), while the other flanks *xrs105* (p2).

To study intermolecular recombination between different plasmids, we took advantage of the AYE strain that contains two different and compatible Rep_3-family plasmids p1ABAYE (p1) and p2ABAYE (p2), both carrying multiple *xrs* (Figure 5). The central regions of *xrs6* (p1) and *xrs105* (p2) are identical, as are those of *xrs107* (p1) and *xrs10* (p2), whereas the central regions of *xrs66* and *xrs38* diverge at one position. We monitored intermolecular recombination events using a PCR-based approach, with pairs of oligonucleotides amplifying the DNA region resulting from intermolecular recombination between *xrs*. Recombination was observed between two sets of *xrs* (*xrs6/105* and *xrs66/38*, Figure 5, lanes 2 and 4) but not for *xrs107/10* (Figure 5, lane 6). Sequencing data showed that recombination occurred within the *xrs* (Supplementary Figure S3), whereas recombination was abrogated in a Δ*xerC* derivative (Figure 5, lanes 7–12). Note that *xrs107* was poorly recognized by XerCD*Ab* (Figure 2), which may explain the lack of recombination. Site-specific recombination was not observed between *xrs* that do not share a closely related central region (Supplementary Figure S3). Therefore, *Ab* plasmids form cointegrated structures by recombination between *xrs* provided closely related central regions. These cointegrates are dynamic and the observed plasmid configuration most likely reflects the most stable genetic structure. Further testing their resolution, as proposed in the Balalovski and Grainge model, would require performing experimental assay stabilizing these cointegrates (20).

We attempted to quantify the ratio of cointegrates using ddPCR (Supplementary Figure S4). Cointegrates formed by *xrs6*–*xrs105* recombination represented 0.25% of the DNA matrix leading to the PCR observed from p2 (Figure 5B, lane 1). Considering that p2 appears ≈1.5 times more abundant than p1, we estimate a rate of cointegrate of 0.45% for p1. Consistent results were obtained for recombination between *xrs38* and *xrs66*, confirming a steady-state rate of 0.1–1% of cointegrate between the two plasmids.

## Discussion

The discovery that some antibiotic resistance genes in *Ab* plasmids are flanked by sequences similar to *xrs* (17) suggested that the Xer system may be involved in the mobility of these genetic structures, promoting the spread of antibiotic resistance in *Ab*. However, it was not clear how mobility occurs, given what was known about the Xer system in model bacteria (2).

The Xer system is highly conserved in bacteria (4), and the few functional studies show that its function in resolving dimeric forms of the chromosome, as well as the manner in which recombination is controlled by FtsK, is also conserved (31,35–38). Consistently, we found that XerC_Ab_ and XerD_Ab_ together recombine *dif_Ab_* site in an FtsK-dependent manner and that *xerC_Ab_* deletion induces a filamentous phenotype like that induced by unresolved dimers in *E. coli*.

Interactions of *Ab* recombinases with the predicted *xrs* were readily detected, but DNA cleavage was dependent on the FtsKγ domain of FtsK_Ab_, showing that recombination is controlled at the cleavage step as in *E. coli*. No recombination leading to deletion was detected between the *xrs* flanking adaptation genes in the AYE’s plasmids. This was expected since these pairs of *xrs* are inverted and do not share the same central region (2,5,6). However, there is no deletion between the *xrs* flanking the *relBE* operon in the pK50b plasmid (K50 strain) (34), although these two *xrs* are directly repeated and share the same central region.

It seems that somehow intramolecular recombination between *xrs* sequences found on *Ab* plasmids is inefficient. This may be because the XerC binding arms of these sequences are far from consensus and are poorly bound by XerC alone.

Taken together, these results make it unlikely that the Xer-driven mobility of genes between plasmids, suggested by sequence analysis, involves excision of a circle of DNA by recombination between the *xrs*.

Rather, our results suggest that Xer-dependent mobility involves plasmid cointegration by recombination between *xrs*. Cointegration of Rep_3 family plasmids in *Ab* has been described in previous work when plasmid encounter is triggered by their artificial transfer, leading to the proposal that mobility may involve plasmid cointegration as an intermediate (19,20,22). We have shown that cointegrates form by Xer recombination between plasmids of the strain AYE during normal growth. This strain contains two plasmids of the Rep_3 family, p1 and p2, each containing several *xrs*. All tested *xrs* are recognized by XerCD and all support catalysis, except *xrs107*. This latter *xrs* diverges in the conserved motif flanking the central regions (ATAA-central region-TTAT), whose sequence determines the binding and cutting activities of XerC and XerD (4). Two pairs of *xrs* appeared to be compatible for recombination: *xrs06* (p1) and *xrs105* (p2), which share a GGTGTA central region, and *xrs66* (p1) and *xrs38* (p2), which share a T/CCGCCA central region. These two pairs of *xrs* recombine to form cointegrates of the p1 and p2 plasmids. This shows that in *Ab*, cointegrates form between plasmids present in the same cell in a process dependent on the XerCD system. This supports the model proposed by Balalovski and Grainge that Xer-driven mobility of *Ab* plasmid genes occurs in two steps: a first recombination between a pair of *xrs* present on each plasmid leads to their cointegration, and a second event between another pair of *xrs* would then resolve the cointegrate and lead to exchange of the *xrs*-flanked genes between the plasmids (20).

Cointegrates of the p1 and p2 plasmids, whether formed by recombination at one pair of *xrs* or the other one, are <1% of the population of either plasmid. This suggests that recombination frequencies are low compared to Xer recombination events resolving dimers of replicons, either plasmids or chromosomes, which occur whenever needed during a cell cycle. In enterobacteria, recombination at plasmid-borne *xrs* is induced by *cis*-elements, DNA sequences and associated proteins (15). These elements limit recombination to intramolecular events (dimer dissociation) and thus should not induce cointegrate formation. However, we did not find any of these *cis-*elements on the plasmids tested here. Rather, recombination at plasmid-borne *xrs* in *Ab* is controlled by FtsK, as is the resolution of chromosome dimer. FtsK restricts recombination in time and space: to the time of cell division (7,39,40); to the chromosomal region opposite the origin of replication (KOPS orientation bias) (11,26); and to the vicinity of the division septum (33,41). This allows for efficient recombination at the chromosome *dif* site but would be expected to result in a much lower frequency of plasmid-borne *xrs*. Indeed, since KOPS are rare on small plasmids (none on the plasmids studied here), they certainly interact infrequently with active septum-bound FtsK (42). This still allows the formation of cointegrates, since FtsK can induce both inter- and intramolecular recombination at plasmid-borne *xrs* (43).

Although we detected recombination at the two pairs of *xrs* present on plasmids p1 and p2, we did not observe exchange of genes located between these *xrs*. This suggests either that cointegrates are preferentially formed and resolved at the same pair of *xrs* or that cointegrates are stable once formed. Gene exchanges due to unequal resolution of the cointegrate may thus be too rare to be detected in our experiments (lasting ∼30 generations). They may also be unselected or even counterselected. According to this view, plasmids p1 and p2, coexisting for a long time, should have reached a stable state, preventing the detection of plasticity under stable conditions. This remains to be tested in future experiments.

This work shows that the Rep_3 plasmids of *Ab* contain multiple active Xer recombination sites and raises the question how these structures can be stably maintained. Indeed, if intermolecular recombination between *xrs* present on different plasmid molecules leads to cointegrate formation, it should also lead to the formation of dimers and higher multimers of the plasmids, which would be deleterious for their stability (14,44). Dimerization of Rep_3 plasmids has not been reported to our knowledge and was not detected in this study. Either recombination between *xrs* present on different copies of the same plasmid is somehow inhibited or multimeric forms of plasmids are efficiently resolved by homologous or site-specific recombination. As these plasmids are devoid of self-encoded site-specific recombinase, they may use the Xer system for this purpose, as described for enterobacterial plasmids (2,45). Interestingly, *xrs38* and *xrs66* (from p2 and p1AYE, respectively) can recombine independently of FtsK, suggesting a role in plasmid dimer resolution. However, we did not find any accessory sequences in the vicinity of these *xrs* that could regulate their activity, as is the case for classical plasmid dimer resolution sites (15). These two sites have very classical XerC and XerD binding arms, but they share a central region that is different from the other *xrs* tested (T/CCGCCA). This central region may be responsible for the FtsK-independent cleavage activity of XerD. This needs to be investigated in future experiments.

Taken together, our results strongly suggest that *Ab* Rep_3 plasmids frequently recombine by Xer recombination, highlighting an original controlled cointegration/resolution mechanism involved in the acquisition and combination of antibiotic resistance genes in this human pathogen.

## Supplementary Material

gkae1255_Supplemental_Files

## Data Availability

The data underlying this article will be shared on reasonable request to the corresponding author.

## Notes

Present address: Rachel Contarin, INTHERES, Université de Toulouse, INRAE, ENVT, Toulouse, France.
