# Supplementary material for: Interplay between the Xer recombination system and the dissemination of antibioresistance in Acinetobacter baumannii
Source: Nucleic Acids Res. 2025 Jan 7;53(1):gkae1255. doi: 10.1093/nar/gkae1255 (PMC11705084; doi:10.1093/nar/gkae1255)
Supplement: gkae1255_Supplemental_Files [file gkae1255_supplemental_files.zip › Blanchais-2024-sup-revision-2.pdf]

Table SUP1		
**Strains**	**genotype**	**Ref**
CP109	LN2666 del (dif) ::Tc , del(lac i), xer :: Gm ref Deghorain 2011	This study
CP1518	LN2666 dif :: Tc :: *difAb-laciQ-difAb* , del(lac i), xerC :: Gm	This study
CP1088	[LN2666: W1485 F-leu thyA thi deoB or C supE rpsl (StR); xerD::frt; xerC::frt]	Fournes *et al.,* 2016
CS20	LN2666 del(RecF)::FRT-Kn-FRT	This study
CB50	LN2666 xerD :: Frt, xerC :: Frt, recF-::Kn	This study
DH5a	F – φ80lacZDM15 D(lacZYA-argF) U169 recA1 endA1 hsdR17 (rk–, mk+) phoA supE44 l – thi–1 gyrA96 relA1	Sambrook et al., 1989
Ab5075-T		Godeux *et al.* 2020
Ab5075ap	AB5075-T *xerC::aacC,sacB*	This study
Ab5075 *xerC-*	AB5075-T *ΔxerC*	This study
AbAYE-T		Godeux *et al.* 2020
AbAYEap	ABAYE-T *xerC::aacC,sacB*	This study
AbAYE *xerC-*	ABAYE-T *ΔxerC*	This study
**Plasmids**	**génotype**	**Ref**
pCP179	pLN135 (Cm), Δdif ::difAb-laciQ-difAb	
pLN1xerCEc	pSC101(Cm), pBAD::xerCEc	This study
pLN1xerCAb	pSC101(Cm), pBAD::xerCAb	This study
pLN1xerCDEc	pSC101(Cm), pBAD::CDEc	This study
pLN1xerCDAb	pSC101(Cm), pBAD::xerCDAb	This study
pLN1xerCDgAb	pSC101(Cm), pBAD::xerCDγAb	This study
pLN1xerCDRQAb	pSC101(Cm), pBAD::xerCD(RQ)Ab	This study
pFX346	pBR322(Amp), dif-Cm-dif	Diagne *et al.* 2011
pROUT25	pFX346, xrs38-*lacIq-xrs*43 (pABV01 like)	This study
pROUT29	pFX346, difAb-lacIq-difAb	This study
pROUT31	pFX346, xrs105-lacIq-xrs38 (p2ABAYE)	This study
pROUT32	pFX346, xrs6-lacIq-xrs38 (p2AB5075)	This study
pROUT26	pET32, pT7::XerCAb	This study
pROUT27	pET32, pT7::XerCAb	This study
pxerDgAb	pET32, pT7::XerDγAb	This study
pxerCAbRQ	pET32, pT7::XerC(RQ)Ab	This study
pxerDAbRQ	pET32, pT7::XerC(RQ)Ab	This study
**Primer**	**sequence**	
DIFAB-TOP	ATCGTGGATCCTGTTCGTATAATGTATATTATGT TAAATTCTAGAGTCGACATCCT	
DIFAB-BOTTOM	AGGATGTCGACTCTAGAATTTAACATAATATAC ATTATACGAACAGGATCCACGAT	
DIFAB-TOP-LEFT	ATCGTGGATCCTGTTCGTATAATG	
DIFAB-TOP-RIGHT	TATATTATGTTAAATTCTAGAGTCGACATCCT	
DIFAB-BOTTOM-LEFT	AGGATGTCGACTCTAGAATTTAACATAATA	
DIFAB-BOTTOM-RIGHT	TACATTATACGAACAGGATCCACGAT	

DIFAB-CY3 (TOP)	CY3-ATCGTGGATCCTGTTCGTATAATGTATATTATGTTAAATTCTAGAGTCGACATCCT
38-T	ATCGTGGATCCACTTCGTATAATCGCCATTATGTTAAATTCTAGAGTCGACATCCT
38-B	AGGATGTCGACTCTAGAATTTAACATAATGGCGATTATACGAAGTGGATCCACGAT
38-T-L	ATCGTGGATCCACTTCGTATAATC
38-T-R	GCCATTATGTTAAATTCTAGAGTCGACATCCT
38-B-L	AGGATGTCGACTCTAGAATTTAACATAATG
38-B-R	GCGATTATACGAAGTGGATCCACGAT
38-T-CY3	CY3-ATCGTGGATCCACTTCGTATAATCGCCATTATGTTAAATTCTAGAGTCGACATCCT
43-B	ATCGTGGATCCAATTAACATAATACACGTTATACGAAATTCTAGAGTCGACATCCT
43-T	AGGATGTCGACTCTAGAATTTCGTATAACGTGTATTATGTTAATTGGATCCACGAT
43-B-R	ATCGTGGATCCAATTAACATAATA
43-B-L	CACGTTATACGAAATTCTAGAGTCGACATCCT
43-T-L	AGGATGTCGACTCTAGAATTTCGTATAACG
43-T-R	TGTATTATGTTAATTGGATCCACGAT
43-B-CY3	CY3-ATCGTGGATCCAATTAACATAATACACGTTATACGAAATTCTAGAGTCGACATCCT
6-T-CY3	CY3-ATCGTGGATCCATTTCGTATAAGGTGTATTATGTTAATTTCTAGAGTCGACATCCT
6-T	ATCGTGGATCCATTTCGTATAAGGTGTATTATGTTAATTTCTAGAGTCGACATCCT
6-B	AGGATGTCGACTCTAGAAATTAACATAATACACCTTATACGAAATGGATCCACGAT
6-T-L	ATCGTGGATCCATTTCGTATAAGG
6-T-R	TGTATTATGTTAATTTCTAGAGTCGACATCCT
6-B-R	AGGATGTCGACTCTAGAAATTAACATAATA
6-B-L	CACCTTATACGAAATGGATCCACGAT
49-T-CY3	CY3-ATCGTGGATCCGCTTCGCATAAGAGATTTTATGTTAAATTCTAGAGTCGACATCCT
49-T	ATCGTGGATCCGCTTCGCATAAGAGATTTTATGTTAAATTCTAGAGTCGACATCCT
49-B	AGGATGTCGACTCTAGAATTTAACATAAAATCTCTTATGCGAAGCGGATCCACGAT
49-T-L	ATCGTGGATCCGCTTCGCATAAGA
49-T-R	GATTTTATGTTAAATTCTAGAGTCGACATCCT
49-B-R	AGGATGTCGACTCTAGAATTTAACATAAAA
49-B-L	TCTCTTATGCGAAGCGGATCCACGAT
10-T-CY3	CY3-ATCGTGGATCCGCTTCGTATAAGAGATTTTATGTTAAATTCTAGAGTCGACATCCT
10-T	ATCGTGGATCCGCTTCGTATAAGAGATTTTATGTTAAATTCTAGAGTCGACATCCT
10-B	AGGATGTCGACTCTAGAATTTAACATAAAATCTCTTATACGAAGCGGATCCACGAT
10-T-L	ATCGTGGATCCGCTTCGTATAAGA
10-T-R	GATTTTATGTTAAATTCTAGAGTCGACATCCT

10-B-R	AGGATGTCGACTCTAGAATTTAACATAAAA
10-B-L	TCTCTTATACGAAGCGGATCCACGAT
105-T-CY3	CY3-ATCGTGGATCCATTTCGTATAAGGTGTATTATGTTAAGTTTCTAGAGTCGACATCCT
105-T	ATCGTGGATCCATTTCGTATAAGGTGTATTATGTTAAGTTTCTAGAGTCGACATCCT
105-B	AGGATGTCGACTCTAGAAACTTAACATAATACACCTTATACGAAATGGATCCACGAT
105-T-L	ATCGTGGATCCATTTCGTATAAGG
105-T-R	TGTATTATGTTAAGTTTCTAGAGTCGACATCCT
105-B-R	AGGATGTCGACTCTAGAAACTTAACATAATA
105-B-L	CACCTTATACGAAATGGATCCACGAT
66-Top-CY3	CY3-ATCGTGGATCCATTTCGTATAACCGCCATTATGTTAAATTCTAGAGTCGACATCCT
66-T	ATCGTGGATCCATTTCGTATAACCGCCATTATGTTAAATTCTAGAGTCGACATCCT
66 B	AGGATGTCGACTCTAGAATTTAACATAATGGCGGTTATACGAAATGGATCCACGAT
66-T-L	ATCGTGGATCCATTTCGTATAACC
66-T-R	GCCATTATGTTAAATTCTAGAGTCGACATCCT
66 B-L	AGGATGTCGACTCTAGAATTTAACATAATG
66 B-R	GCGGTTATACGAAATGGATCCACGAT
107-T-CY3	CY3-ATCGTGGATCCATTTCGCATATGAGATTTTGTATTAAATTCTAGAGTCGACATCCT
107-T	ATCGTGGATCCATTTCGCATATGAGATTTTGTATTAAATTCTAGAGTCGACATCCT
107-B	AGGATGTCGACTCTAGAATTTAATACAAAATCTCATATGCGAAATGGATCCACGAT
107-T-L	ATCGTGGATCCATTTCGCATATGA
107-T-R	GATTTTGTATTAAATTCTAGAGTCGACATCCT
107-B-R	AGGATGTCGACTCTAGAATTTAATACAAAA
107-B-L	TCTCATATGCGAAATGGATCCACGAT
Cassette-difAb-difAb-TOP	CCGGATCCTGTTCGTATAATGTATATTATGTTAAATCTAGAGTCGACCTGCAGTGTTCGTATAATGTATATTATGTTAAATGCATGCGG
Cassette-difAb-difAb-Bottom	CCGCATGCATTTAACATAATATACATTATACGAACACTGCAGGTCGACTCTAGATTTAACATAATATACATTATACGAACAGGATCCGG
Cassette-PABV01-T	CCGGATCCACTTCGTATAATCGCCATTATGTTAAATCTAGAGTCGACCTGCAGAATTAACATAATACACGTTATACGAAATGCATGCGG
Cassette-PABV01-B	CCGCATGCATTTCGTATAACGTGTATTATGTTAATTCTGCAGGTCGACTCTAGATTTAACATAATGGCGATTATACGAAGTGGATCCGG
Cassette-p2ABAYE-T	CCGGATCCATTTCGTATAAGGTGTATTATGTTAAGTCTAGAGTCGACCTGCAGATTTAACATAATGGCGATTATACGAAGTGCATGCGG
Cassette-p2ABAYE-B	CCGCATGCACTTCGTATAATCGCCATTATGTTAAATCTGCAGGTCGACTCTAGACTTAACATAATACACCTTATACGAAATGGATCCGG
Cassette-pAB5075-T	CCGGATCCATTTCGTATAAGGTGTATTATGTTAATTCTAGAGTCGACCTGCAGATTTAACATAATGGCGATTATACGAAGTGCATGCGG

Cassette-pAB5075-B	CCGCATGCACTTCGTATAATCGCCATTATGTTAAATCTGCAGGTCGACTCTAGAATTAACATAATACACCTTATACGAAATGGATCCGG
74	TGTTGCCGAAGAGCCAGAAG
75	ggaacttcgaagcagctccagcctacacaatcAGCTCACAAAACTCAAGGAAGC
76	gattgtgtaggctggagctgcttcgaagttccatcACCCATCACATATACCTGCCGTTCAC
77	gccatggtccatatgaatatcctccttagttcTCATGAGCTCAGCCAATCGACTGG
78	gaactaaggaggatattcatatggaccatggcAGCCACCAAGCATTAAGCC
79	GCGTTCAAATCACCAACTAACC
80	cttggtggctAGCTCACAAAACTCAAGGAAGC
81	tttgtgagctAGCCACCAAGCATTAAGCC
O1	TTGGTCTAGAGGAAGGAGAACGCGTATGACCGATTTACAC
O2	TTGGATGCATGGCGCATTATTTCCCCCGTTTGG
O3	TTGGATGCATGGAAGGAGATCTGGCATGAAACAGGATCTGGCACGC
O4	TTGGATGCATGGTCACGCCCGCGGGTGATGC
O5	TTGGTCTAGAGGAAGGAGAACGCGTATGGAGAGCAATTTGGATTTTGC
O6	TTGGATGCATCCTAATGCTTGGTGGCTCGTGG
O7	TTGGATGCATGGAAGGAGATCTGGCATGATAAATAAAAAACCGCGTATTCC
O8	TTGGATGCATCCTAACCCCTAGGGTGATGTTTTTC
94	AGACTCTTCTTCTTCCACCAC
95	GATGTTACCCAACAAGGGAAG
101	ATGAAGTCAAAAATGGGAGCCT
103	CGTCACTTTAGCCAATTTTCCAATC
105	AGAGATCCGAACACTTTGGAG
117	ttctgcaacctgtctgatgc
119	tcctacgactccaaaatcctg
120	tgaaggaacaggaaaaccaga
121	tccatcacttggcataactca
141	tagcgtggaacgtgaaaagtc
142	ccgtaggtaatgctcccaaa
143	ccagagggtatgtgggctaa
145	ggtaggggaatactccctcaaa
146	ggcaagaagtatcaaaactgctc
148	atagggtaagctgcgtcaaaac

A

Assay	Colonies: Blue	Total	%	Recombination rate %
1	46	440	10	10
2	13	136	10	
3	246	2387	10	

B

Assay	Colonies: Blue	Total	%	Recombination rate %
1	46	87	53	47
2	209	498	41	
3	232	507	46	

Blanchais et al., Figure Sup1

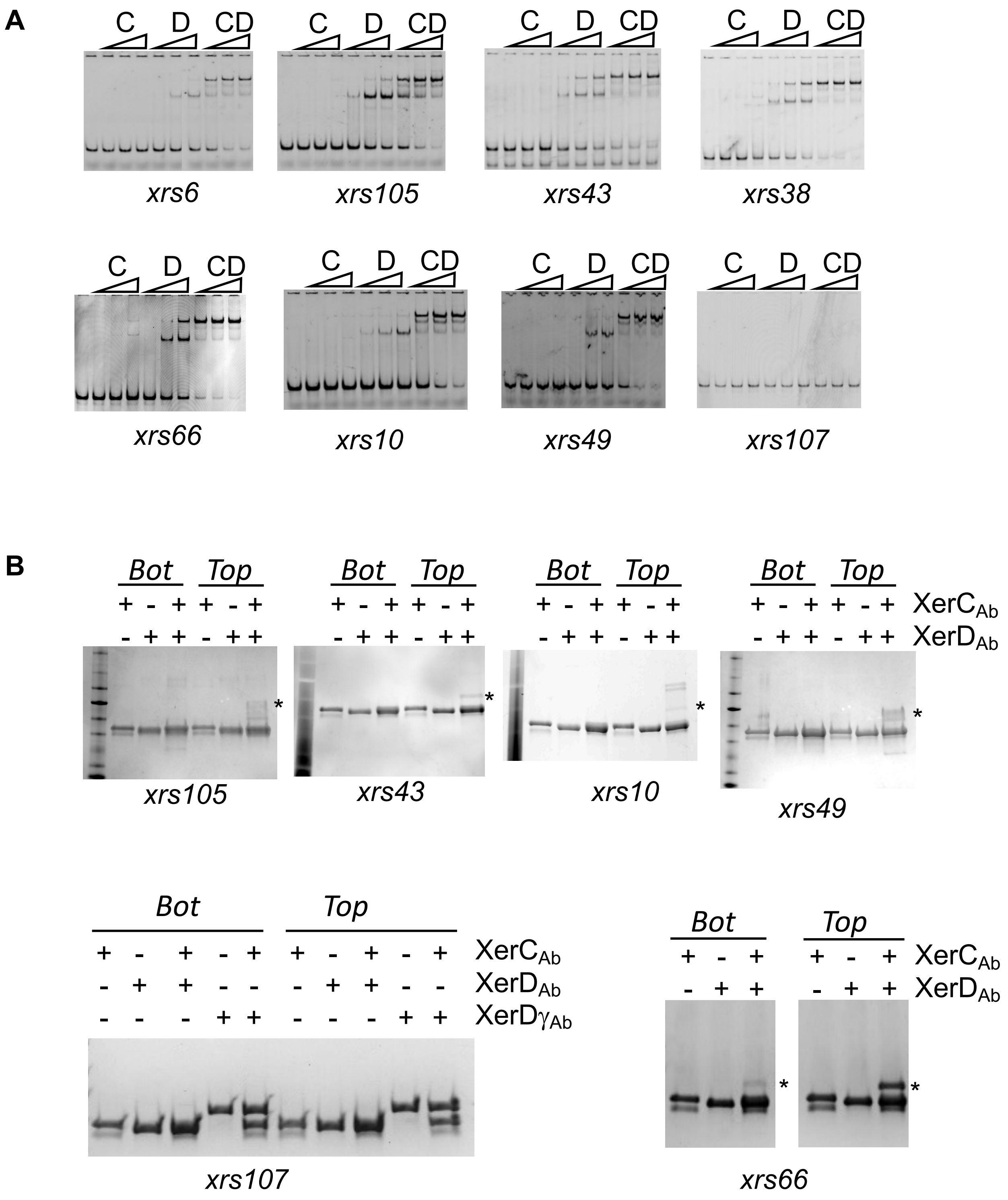

A

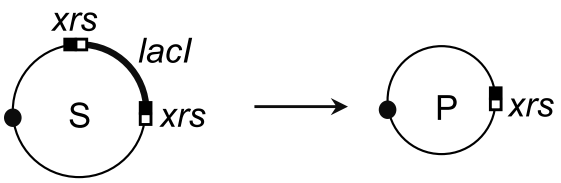

B

xrs-pK50$_a$: atttcgtataa cgtgta ttatgttattt
xrs-pK50$_b$: acttcgtataa cgtgta ttatgttaatt

C

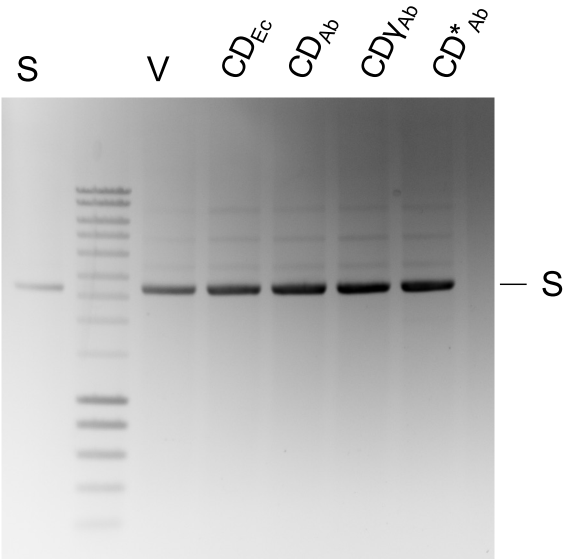

Blanchais et al., Figure Sup3

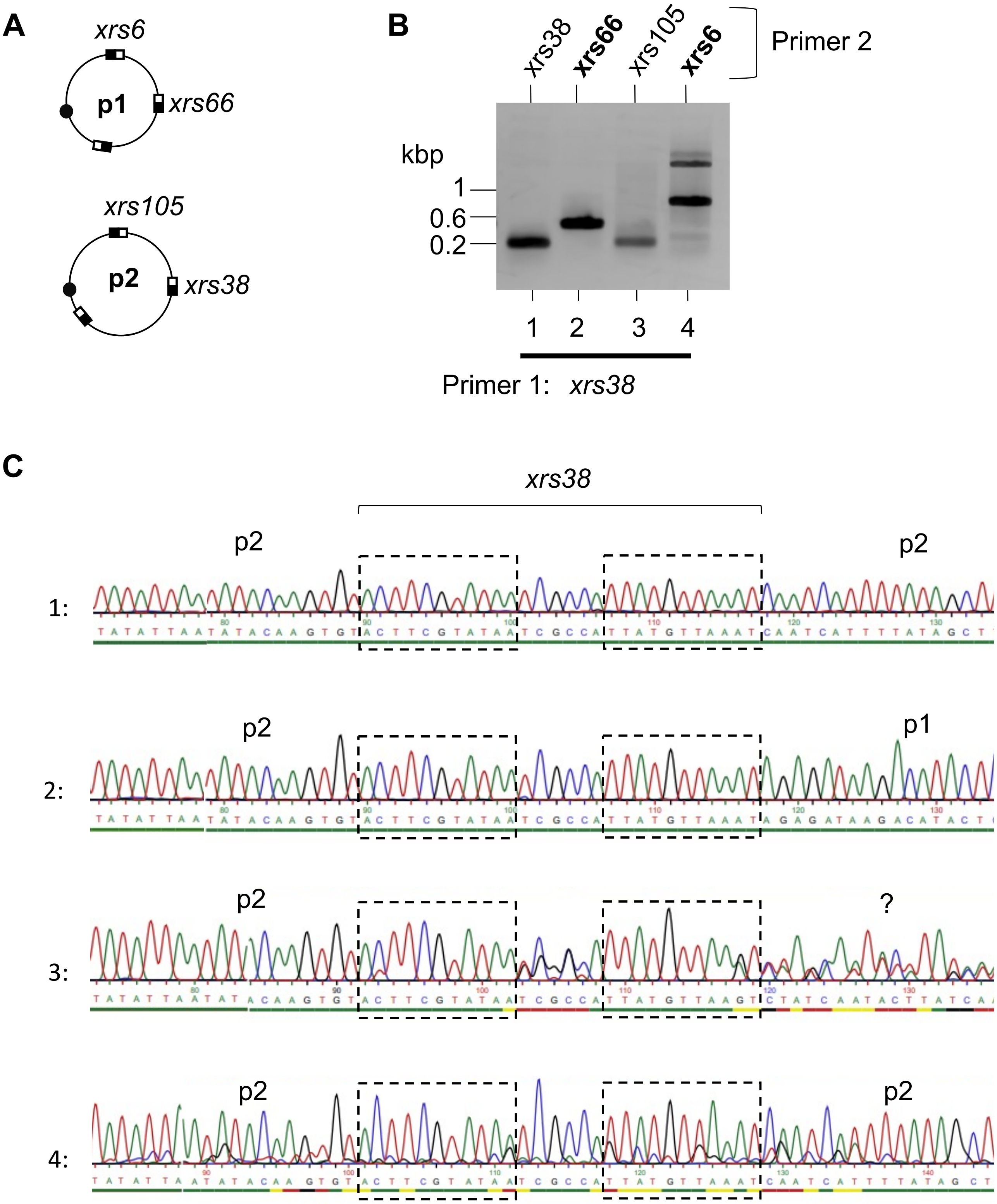

Blanchais et al., Figure Sup4

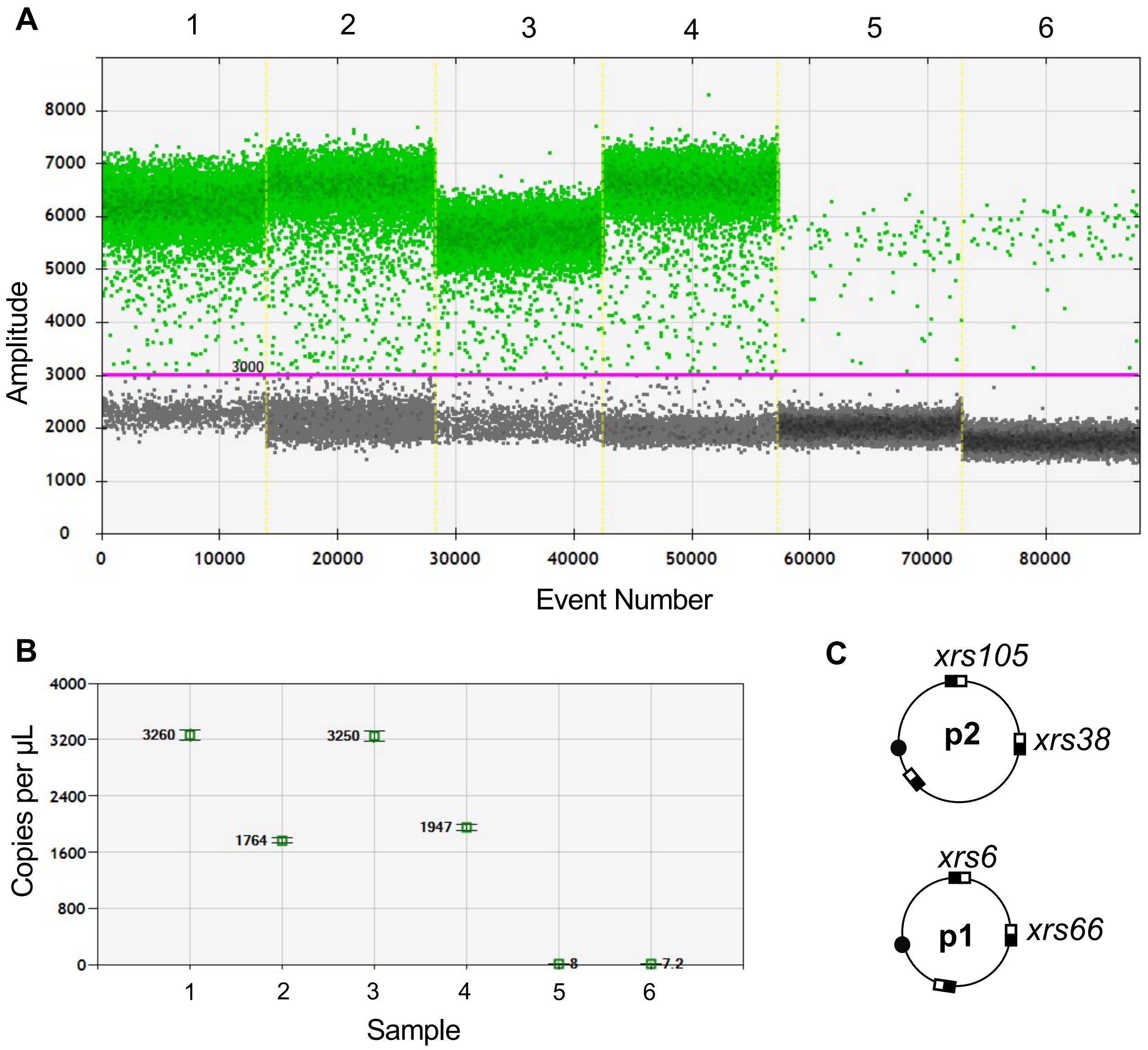

D

Reaction	Targeted *xrs*	Primers	Amplicon size (bp)	Copies/µL	Copies/20µL	Positives	Negatives	Accepted Droplets
1	*xrs105*	141-142	200	3260	65200	13127	876	14003
2	*xrs6*	143-144	236	1764	35280	11142	3201	14343
3	*xrs38*	145-146	200	3250	65000	13300	900	14200
4	*xrs66*	147-148	268	1947	38940	11979	2829	14808
5	*xrs105/6*	142-143	165	8	160	106	15507	15613
6	*xrs38/66*	145-148	180	7.2	144	92	14893	14985

Table Sup2: Deletion rate of a *dif*$_{Ab}$-*lacI*-*dif*$_{Ab}$ cassette inserted into the *E. coli* chromosome at *dif* (Deghorain et al. 2011). A) The strain is deleted for *xerC* and complemented with pLN1::*xerCD*$_{Ab}$. B) The strain is deleted for *xerC* and complemented with pLN1::xerCDγ_{Ab}

Figure Sup 1: Activity of the *xrs* of *Ab (summarized in Figure 3*. A) EMSA of *xrs* by XerCDAb . The DNA fragment (*xrs*) is 56 bp long and is 5'-CY3. XerCAb (mM) = 0; 0.48; 1.2 and 2.4. XerDAb (mM) = 0; 0.64; 1.6 and 3.2. B) SDS-PAGE analysis of cleavage assays (top diagram). DNA molecules (*xrs* top or bottom nicked) are 10mM while proteins are 2 mM. Covalent products formed between DNA and proteins are indicated (*).

Figure Sup2: *xrs*-cassette deletion. **A)** The XerCD recombinase, when produced, should delete the *lacI* gene from the substrate plasmid (S), resulting in a deleted plasmid (P) and a non-replicative circle containing *lacI*, which is lost during divisions. **B)** Sequences of the two xrs found on pk50. They are directly repeated on this plasmid.**C)** Gel electrophoresis of the plasmid extraction after overnight culture of different strains transformed with a substrate plasmid containing a *xrspK50a-lacI-xrspK50b* cassette: S, substrate plasmid used to transform cells; V, plasmids extracted from a strain not expressing any *xer* genes; CD$_{Ec}$, plasmids extracted from a strain expressing *xerCD* of *E. coli ;* CD$_{Ab}$, plasmids extracted from a strain expressing *xerCD* of *Ab;* CDγ_{Ab}, plasmids extracted from a strain expressing *xerC*$_{Ab}$ and a fusion between *xerD*$_{Ab}$ and the g domain *of ftsK*$_{Ab}$. Substrate (S) plasmid is indicated, no product is observed on the gel.

Figure Sup3: Xer-dependent plasmid recombination involving *xrs38* (p2ABAYE). A) Simplified genetic map of p1ABAYE (p1) and p2ABAYE (p2). **B)** Gel electrophoresis analysis of the PCR reaction performed on plasmid DNA extracted from ABAYE strains. The first primer hybridizes upstream of the XerC arm of *xrs38*. The second primer hybridizes upstream of the XerD arm of *xrs105* or *xrs6* or *xrs38* or *xrs66*. **C)** Sequencing revealed that the PCR product of lane 1 corresponds to a non-recombined p2ABAYE and that the PCR product of lane 2 corresponds to recombination between p1 and p2ABAYE within *xrs38.* The PCR product obtained in lanes 3 and 4 does not correspond to a recombination between *xrs38* and any of the other tested *xrs.*

Figure Sup 4: ddPCR for recombination quantification. **A)** Each column represents single ddPCR amplification. Green dots represent positive droplets while black dots represent negative droplets. Column 1 corresponds to the amplification of *xrs105,* column 2 corresponds to the amplification of *xrs6,* column 3 corresponds to the amplification of *xrs38,* column 4 corresponds to the amplification of *xrs66,* column 5 corresponds to the amplification of a fusion between *xrs105* and *xrs6* (recombination between p1 and p2), column 6 corresponds to the amplification of a fusion between *xrs38* and *xrs66* (recombination between p1 and p2). **B)** The estimated copy number of each DNA matrix molecule is shown. **C) S**implified genetic map of p1ABAYE (p1) and p2ABAYE (p2). **D)** Summary of the data obtained from these 6 different ddPCR reactions. The estimated copy number of the matrix molecules (either recombined or not) is calculated using the Biorad QuantaSoft droplet reader software. The recombination ratio (see text) is calculated by dividing

the number of recombined molecules by the sum of recombined and non-recombined molecules.
