# Supplementary material for: Interplay between the Xer recombination system and the dissemination of antibioresistance in Acinetobacter baumannii
Source: Nucleic Acids Res. 2025 Jan 7;53(1):gkae1255. doi: 10.1093/nar/gkae1255 (PMC11705084; doi:10.1093/nar/gkae1255)
Supplement: gkae1255_Supplemental_Files [file gkae1255_supplemental_files.zip › Blanchais-Table-Sup1-revision-2-title-caption.pdf]

Table SUP1: Biological material used in this study.
Bacterial strains used in this study (name, genotype, reference). Plasmids used in this study (name, genotype, reference). Oligonucleotides used in this study (name, sequences).
